# Computational fluid dynamic simulations for dispersion of nanoparticles in a magnetohydrodynamic liquid: a Galerkin finite element method

**DOI:** 10.1039/c8ra03825b

**Published:** 2018-11-14

**Authors:** M. Nawaz, Shafia Rana, Imran Haider Qureshi

**Affiliations:** Department of Applied Mathematics & Statistics, Institute of Space Technology Islamabad 44000 Pakistan nawaz_d2006@yahoo.com

## Abstract

This investigation studies the effects of the thermo-physical properties of four types of nano-metallic particles on the thermo-physical properties of radiative fluid in the presence of buoyant forces and Joule heating (ohmic dissipation). The Galerkin finite element algorithm is used to perform computations and simulated results are displayed in order to analyze the behavior of velocity and temperature of copper, silver, titanium dioxide and aluminum oxide-nanofluids. All the simulations are performed with *η*_max_ = 6 computational tolerance 10^−6^ for 200 elemental discretizations. Due to the dispersion of nano-sized particles in the base fluid, an increase in the thermal conduction is noticed. This study also predicts future improvements in the thermal systems. Due to magnetic field and fluid flow interaction, the electrical energy converts into heat. This is undesirable in many thermal systems. Therefore, control of Joule heating in the design of thermos systems is necessary. However, this dissipation of heat may be desirable in some biological fluid flows. An increase in energy losses is noted as magnetic intensity is increased.

## Abstract 2

This investigation studies the effects of the thermo-physical properties of four types of nano-metallic particles on the thermo-physical properties of radiative fluid in the presence of buoyant forces and Joule heating (ohmic dissipation).

## Graphical abstract

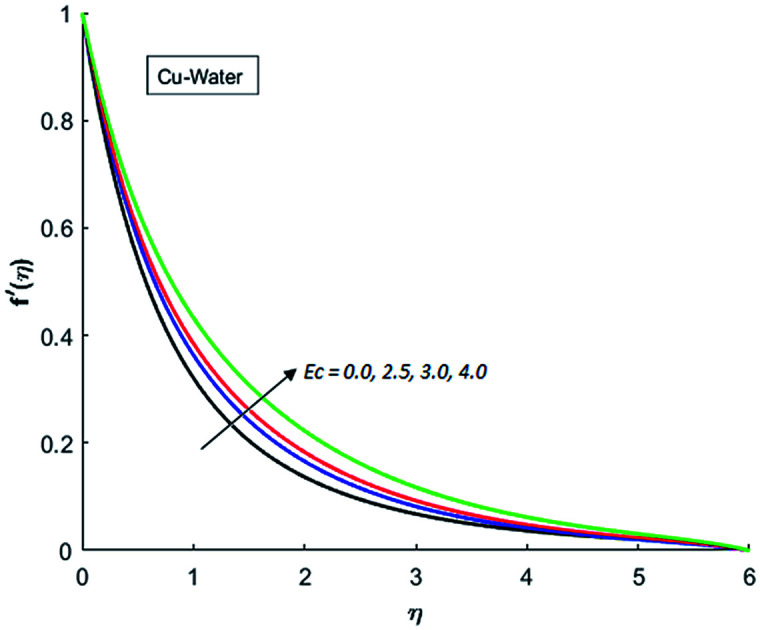

## 1 Introduction

Technologists and engineers have a major concern in enhancing the efficiency of thermal systems like hydronic heating and cooling in buildings, heating and cooling processes of transportation in the petro-chemical industry, pulp and textile manufacturing *etc.*^1^ Several methods to enhance the efficiency of thermal systems have been used for this purpose. These methods include active methods and passive methods.^2^ As mentioned in ref. 2, active methods include external agents like a mechanical input or magnetic field *etc.* whereas passive methods include treated surfaces, insert extended surfaces, boiling, condensation, twisted tape, wire coils^2^*etc.* Combinations of active and passive methods are called compound methods. Although, the above mentioned methods are very effective and have been used for the enhancement of heat transfer, recent advancements in technology have opened the doors to new techniques and methods. One of these methods is the dispersion of nano-metallic particles in pure liquid. This inclusion of particles increases the thermal conductivity of the resulting mixture. Consequently, rate of heat transfer is enhanced. Several theoretical studies on this technique are published. For example Masuda *et al.*^3^ confirmed that the dispersion of ultrafine particles in the base fluid increases its ability to conduct more heat as compared to the pure fluid. Although this work reconfirms the enhancement in the process of transfer of heat due to inclusion of nanoparticles in liquids, this analysis is carried out in a limiting sense *i.e.* Joule heating, thermal radiation, buoyancy effects and heat generation are not considered. The work by Buongiorno^4^ introduced some empirical models for the thermophysical properties of nanofluids and formulated the mathematical relationships between the physical properties of solid particles, pure fluid and mixtures of pure fluid and nano-particles create a potential for theoretical studies on transport of heat by liquid as a coolant containing metal particles of very small size, but this work does not consider Joule heating and buoyancy effects. Transfer of heat in nanofluids over a stretching surface was studied by Khan and Pop.^5^ They investigated thermophoretic and Brownian motion in the flow of nanofluids. However, this work does not consider the heating generation and Joule heating effects simultaneously. Nadeem *et al.*^6^ analyzed the effects of Brownian motion and thermophoresis in the flow of Maxwell fluid. In fact this work does not consider the inclusion of nano-particles rather than thermophoresis and Brownian motion. Das *et al.*^7^ numerically investigated the effects of different types of nano-particles on the entropy generation of MHD flow over convective by heat surface boundary conditions. Although this work considers more than one effect simultaneously but it does not consider Joule heating, thermal radiation, buoyancy and heat generation effects simultaneously. The effect of space dependent magnetic field on free convection flow of Fe_3_O_4_–water nanofluid was studied by Sheikholeslami and Rashidi.^8^ It is important to mention that dispersion of Fe_3_O_4_ nano-particles in the water is considered *i.e.* Cu, Ag, Al_2_O_3_ and TiO_2_ are not considered. In another study, Rashidi *et al.*^9^ investigated the behavior of nano-particles on the thermal conductivity of the base fluid through Lie group approach but heat generation, Joule heating and buoyancy force are not taken into account. Nadeem and Saleem^10^ studied mixed convection flow of nanofluid over a rotating cone in the presence of magnetic fluid. Nawaz and Hayat^11^ studied heat transfer characteristics in an axisymmetric flow of nanofluid over a radially stretching surface. Nawaz and Zubair^12^ analyzed the effects of different types of nano-particles in the flow of blood over a surface moving with space dependent velocity. This work considers only two types of nano-particles (Cu and Ag). Other than this, convective type boundary condition and the entropy generation are not considered in this study.^12^ Ahmed *et al.*^13^ studied the effects of the shape of nanoparticles on mixed convection flow over a disk rotating with time dependent angular velocity. However, this work does not consider Joule heating, heat generation and buoyancy effects simultaneously. These effects will be considered in the present work.

There are various models (empirical formulae) which describe correlations between viscosities of the base fluid and metallic nano-particles and effective viscosities of nanofluids. These models include Einstein model,^14^ Brinkman model,^15^ Batchelor model,^16^ Graham model,^17^ model adopted by Wang *et al.*,^18^ model of Masoumi *et al.*^19^ Einstein model is valid for very low volume fraction (volume fraction < 0.002) and does not consider Brownian motion of nano-particles. Brinkman model is the modified form of the Einstein model and valid for average volume fraction whereas Batchelor model is the modification of Einstein model and considers Brownian motion of nano-particles.^20^ The model used by Wang *et al.*^18^ expresses the effective viscosity as a quadratic function of volume fraction. The model used by Masoumi *et al.*^19^ involves Brownian motion effects. It is also important to note that the models discussed in ref. 14–19 give correlations of effective viscosities. Studies on nanofluid show that dispersion of nano-particles impact thermal conductivity. Therefore, different correlations for effective thermal conductivity proposed. Detailed review on analytical models of effective thermal conductivity is given in ref. 20. It is noted that studies^14–20^ do not consider model of effective electrical conductivity of nano-fluid. As the present work considers magnetohydrodynamic flow of nanofluid and model for effective electrical conductivity is unavoidable. The correlations for effective electrical conductivity, effective thermal conductivity and effective viscosity are used by Das *et al.*^7^ The model used by Das *et al.*^7^ had dual characteristics of effective thermal viscosity and effective thermal conductivity as well as analytical model for effective electrical conductivity. This model is given by1
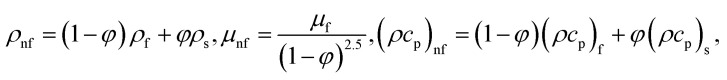
2
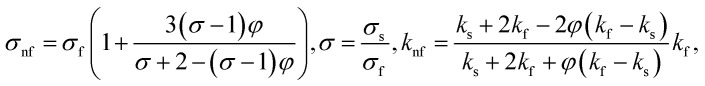
where *ρ*, *k*, *σ*, *φ* and *c*_p_, respectively, are density, thermal conductivity, electrical conductivity, volume fraction and specific heat. The subscripts f, nf and s stands for fluid, nanofluid and solid particles (nano-particles) respectively.

Minimization of the entropy generation in the thermal system is a major concern as wastage of energy causes a great disorder. Therefore, the control of the entropy generation during the heat transfer has been investigated extensively in the last few years. Bejan^21^ was first to work on the minimization of the entropy generation. After his work on the entropy generation, several studies have been published. But, here some recent investigations are described. For instance, Bhatti *et al.*^22^ investigated the effects of magnetic field on the entropy generation of nonlinear transport of heat and mass in the boundary layer flow. Numerical investigation of the entropy generation during the heat transfer in the cavity flow was carried out by Armaghani *et al.*^23^ Vincenzo *et al.*^24^ analyzed the effects of the entropy generation due to temperature difference and viscous losses/friction loses in the flow.

The aim of this work is three fold. First, to study heat transfer enhancement in nanofluids in the presence of applied magnetic field, buoyancy force, thermal radiation and heat generation/absorption using correlation of effective electrical conductivity together with the correlations of effective thermal conductivity and effective viscosity based volume fraction and second is to investigate the effects of dispersion of nano-particles on entropy generation whereas third one is to implement finite element method to two-dimensional hydrothermal flow in the presence of buoyancy force and electromagnetic radiation.

## 2 Problem description

### 2.1 Physical situation

We consider the enhancement of heat transfer in water through four types of nano-particles (Cu, Ag, Al_2_O_3_ and TiO_2_) in an incompressible flow of an electrically conducting fluid over a vertical stretching sheet with space and time dependent velocity *U*_w_(*x*,*t*) = *ax*/(1 − *ct*). A constant magnetic field [0,*B*_0_,0] is applied along *y*-axis normal to the sheet. The variation of the temperature of sheet is due to the variation of hot fluid occupying half space *y* < 0. The temperature of hot fluid below the sheet is varying as *T*_w_(*x*,*t*) = *T*_∞_ + *ax*/(1 − *ct*)^2^ where *T*_∞_ is the ambient temperature, *a* and *c* are constants. There is no applied electric field and the effects of polarization and induced magnetic field are negligible. Thermo-physical properties (viscosity, density, thermal conductivity, specific heat *etc.*) are constant. The transport of heat nanofluid (occupying half space *y* > 0) is due to convection from the hot fluid (occupying half space *y* < 0) of temperature *T*_w_(*x*,*t*) = *T*_∞_ + *ax*/(1 − *ct*)^2^. The buoyant force under Boussinesq approximation is significant.

### 2.2 Governing boundary layer equations

Applying boundary layer approximation to full two-dimensional conservation laws, one obtains the following boundary layer equations3
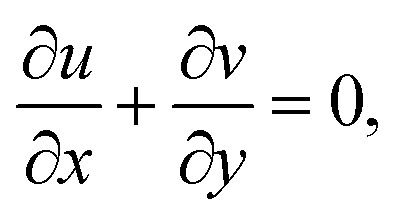
4
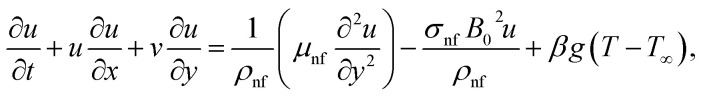
5
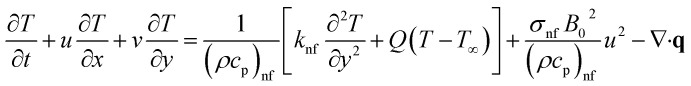
where (*u*, *v*) are the velocity component, *β* is the volumetric expansion coefficient, *Q* = *Q*_0_(1 − *ct*)^−1^ is the heat generation/absorption coefficient, *T* is the temperature of the fluid and **q** is the radiative heat flux vector which is defined by Stefan–Boltzmann law **q** = −(4σ*/3k*)∇(*T*^4^ − *T*_∞_^4^), where σ*is the Stefan–Boltzmann constant and k* is the mean absorption coefficient.

The associated conditions are

6 *u* = *v* = 0,  *T* = *T*_∞_, ∀ *x*, *y*, *t* < 0

7
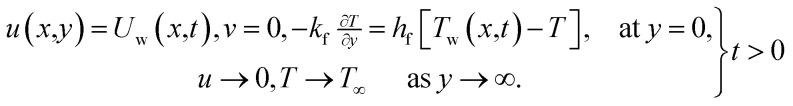


### 2.3 Dimensional analysis

In view of the importance of the results obtained from the dimensionless form of conservation equations, the following transformations are introduced8
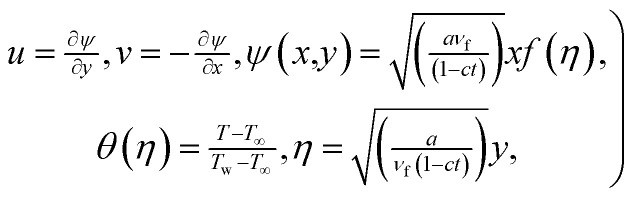
where *ψ*(*x*,*y*) is the stream function, *f*(*η*) and *θ*(*η*) is the dimensionless form of stream function and temperature, *η* is independent similarity variable.

The continuity eqn (3) is identically satisfied and eqn (4) and (5) and conditions are reduced to9
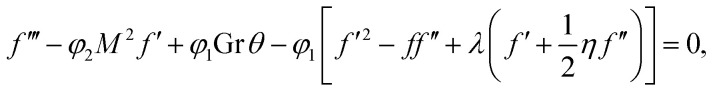

10 *f*(0) = 0, *f*′(0) = 1, *f*′ → 0,

11
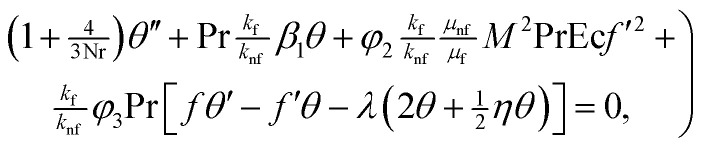

12 *θ*′(0) = −Bi(1 − *θ*(0)), *θ* → 0 as *η* →∞,

where13
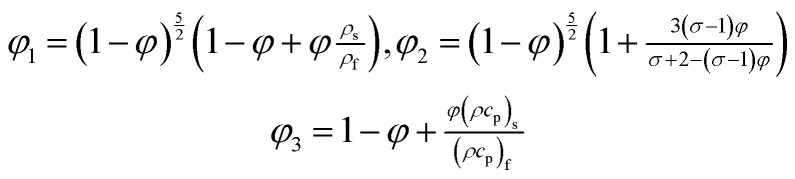
and14
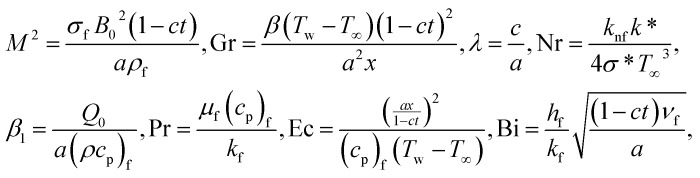
are, respectively, the Hartmann number, the Grashof number, the unsteadiness parameter, the radiation parameter, heat generation/absorption parameter, the Prandtl number, the Eckert number and the Biot number. The prescribed wall temperature case can be recovered as Bi → ∞. Also note that *φ* = 0 is the case when fluid is pure and nano-particles are not dispersed, [the case of Butt and Ali^25^ and for Gr = 0,  Nr = 0 and Ec = 0, the problem reduces to the case of Das *et al.*^7^ with heat generation/absorption. The case of *M*^2^ = 0, *λ* = 0, *φ* = 0 and Bi → ∞ is also considered by Abolbashari *et al.*^26^ and Das *et al.*^7^. The numerical values of thermo-physical properties used in this study are (Table 1).

**Table 1:** Thermo-physical properties of water and nanoparticles^7^

Physical property	Base fluid	Cu	Ag	Al_2_O_3_	TiO_2_
*ρ*/(kg m^−3^)	997.1	8933	10 500	3970	4250
*c*_p_/(J kg^−1^ K^−1^)	4179	385	235	765	686.2
*k*/(W m^−1^ K^−1^)	0.13	401	429	40	8.9538
*φ*	0.00	0.05	0.10	0.15	0.20
*σ*/(s m^−1^)	5.5 × 10^−5^	59.6 × 10^6^	6.6 × 10^−7^	35 × 10^6^	2.6 × 10^6^

## 3 Galerkin finite element formulation

Following studies^12,27–29^ weighted residual approximations (WRA) for the system defined in eqn (9)–(12) are given below
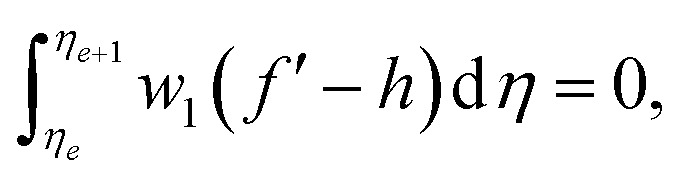

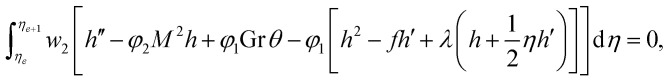

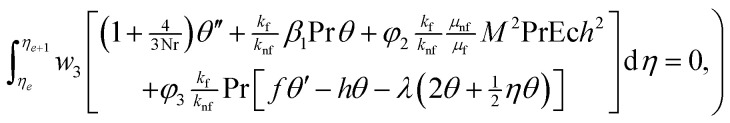
where *f*′ = *h*, the dependent variables are approximated in term of unknown nodal values in the following way
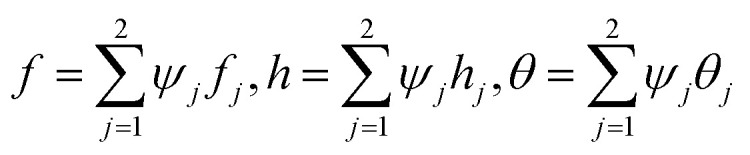
where *w*_1_, *w*_2_, *w*_3_ = *w*_*i*_ are weight functions. For Galerkin approach where *w*_*i*_ = *ψ*_*i*_.

### 3.1 Computation for stiffness matrix

Using the Galerkin finite element scheme, the following elements of stiffness matrix are calculated
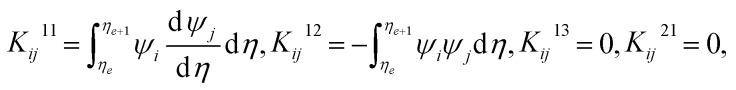

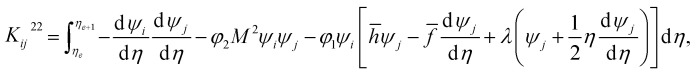

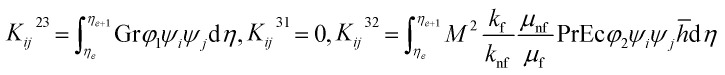

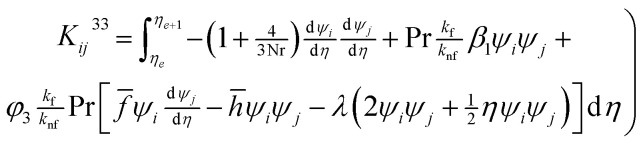

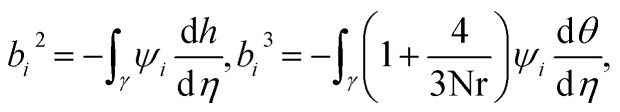
where *h̄* = *ψ*_*j*_*h̄*_*j*_ and *f̄* = *ψ*_*j*_*f̄*_*j*_. *h̄*_*j*_ and *f̄*_*j*_ are nodal values at previous iteration.

## 4 Results and discussion

Galerkin finite element algorithm is implemented to study the effects of thermo-physical properties of nano-sized metallic particles on unsteady two dimensional flows in the presence of buoyant force, thermal radiation and Joule heating. Non-linear stiffness matrix is linearized using Picards linearization scheme and system of algebraic equations are solved iteratively with tolerance 10^−5^. Several numerical experiments are done to search *η*_max_ and grid independent studied is also carried out. Through extensive experiments, we have noted that the computed results converges with tolerance 10^−5^ when *η*_max_ = 6 and domain [0,*η*_max_] is discretized into 200 elements.

### 4.1 Velocity profiles

**Fig. 1:**
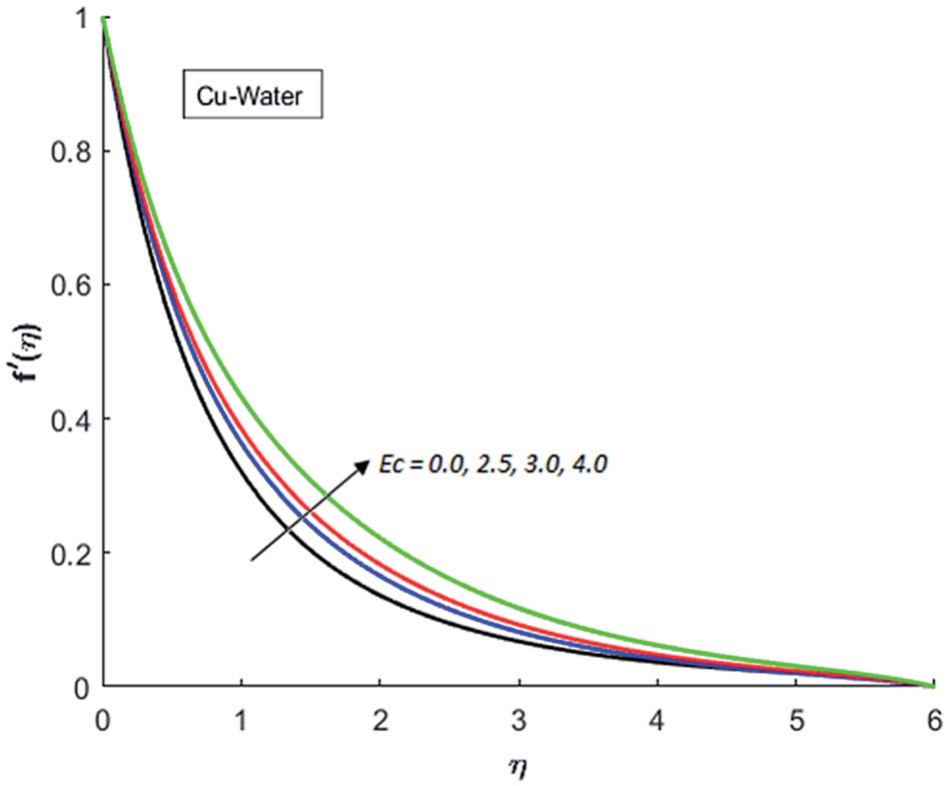
Velocity distribution for various value of Ec when *M* = 0.5, Pr = 0.3, Gr = 5, Nr = 0.2 and *λ* = 1.

**Fig. 2:**
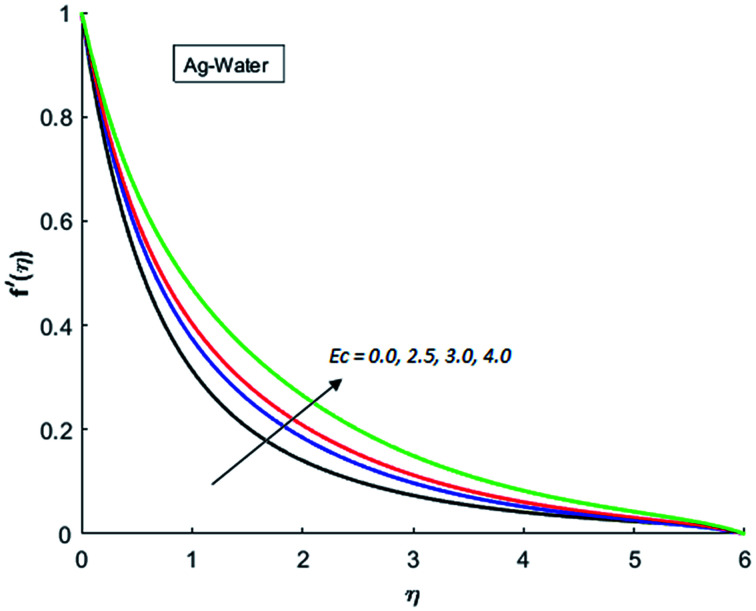
Velocity distribution for various value of Ec when *M* = 0.5, Pr = 0.3, Gr = 5, Nr = 0.2 and *λ* = 1.

**Fig. 3:**
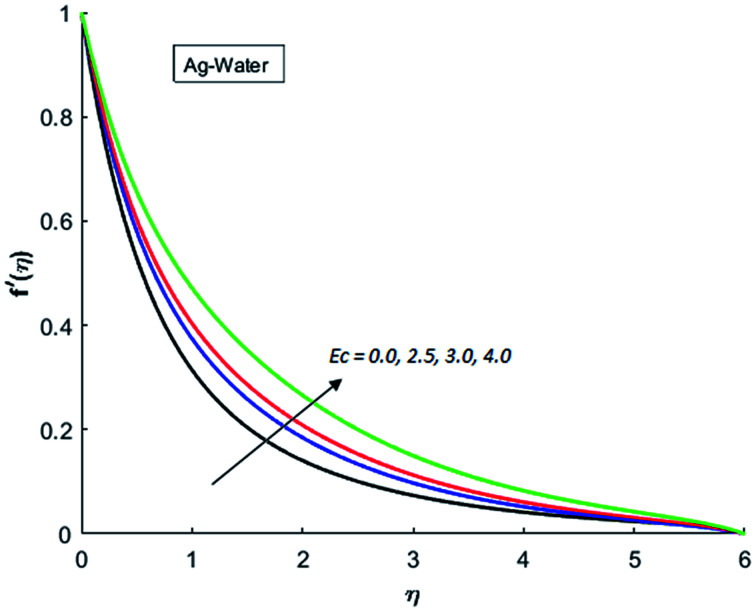
Velocity distribution for various value of Ec when *M* = 0.5, Pr = 0.3, Gr = 5, Nr = 0.2 and *λ* = 1.

**Fig. 4:**
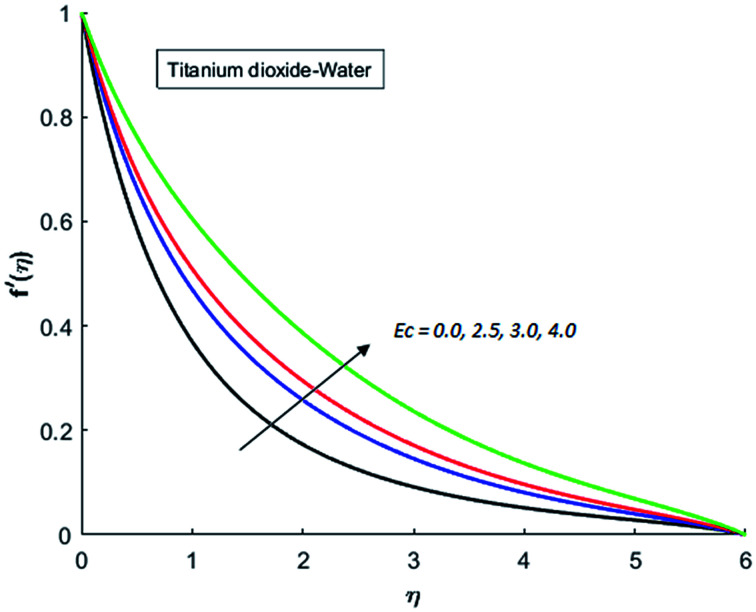
Velocity distribution for various value of Ec when *M* = 0.5, Pr = 0.3, Gr = 5, Nr = 0.2 and *λ* = 1.

**Fig. 5:**
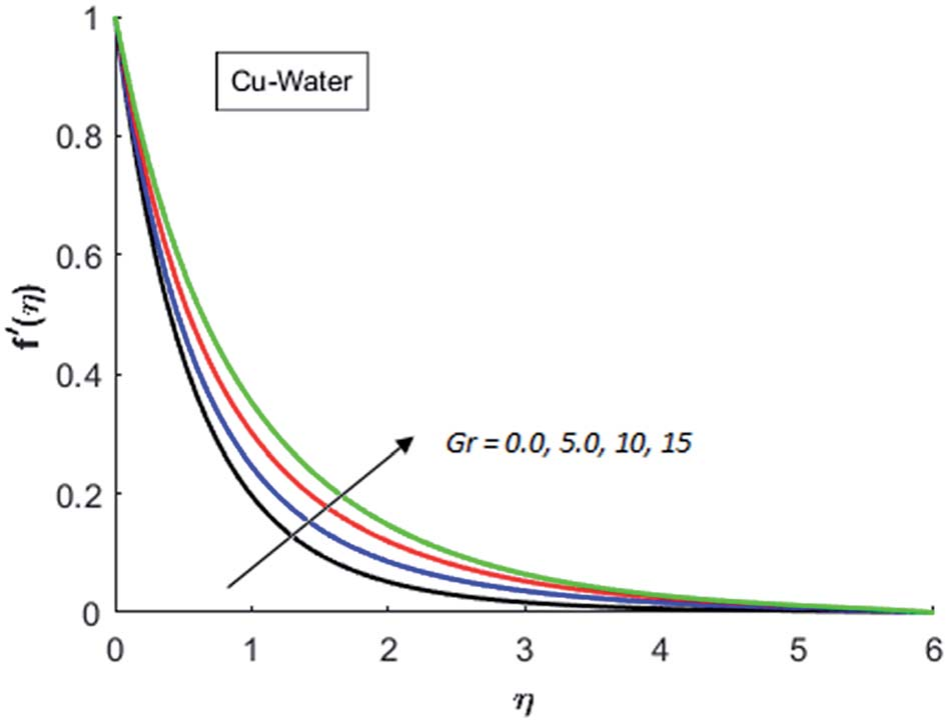
Velocity distribution for various value of Gr when *M* = 1, Pr = 0.3, Ec = 0.1, Nr = 0.2 and *λ* = 1.

**Fig. 6:**
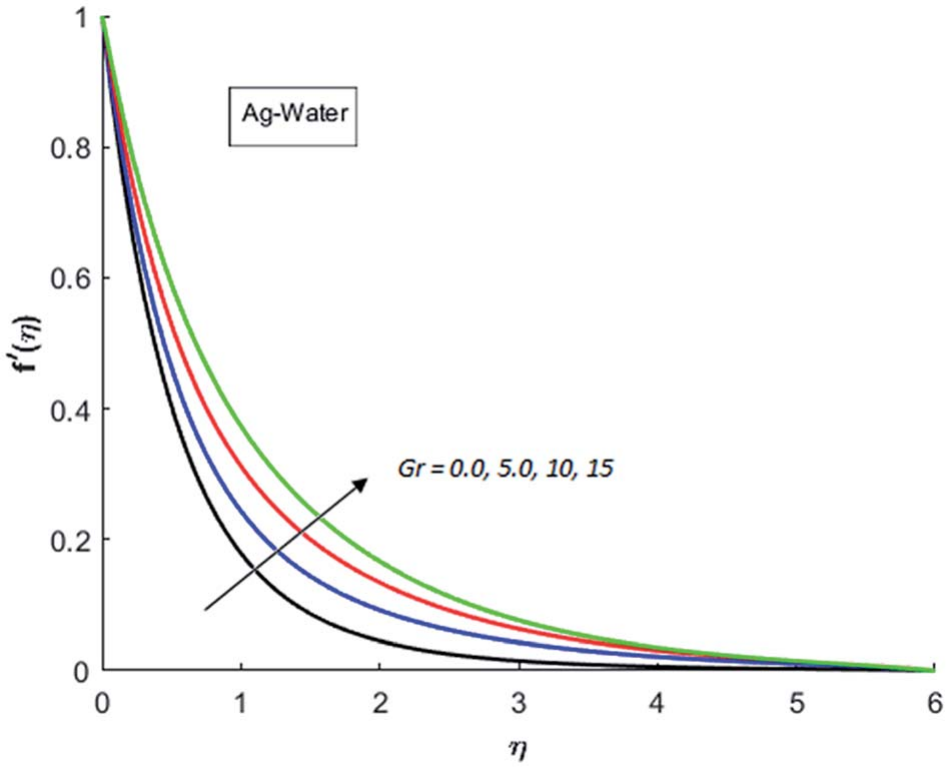
Velocity distribution for various value of Gr when *M* = 1, Pr = 0.3, Ec = 0.1, Nr = 0.2 and *λ* = 1.

**Fig. 7:**
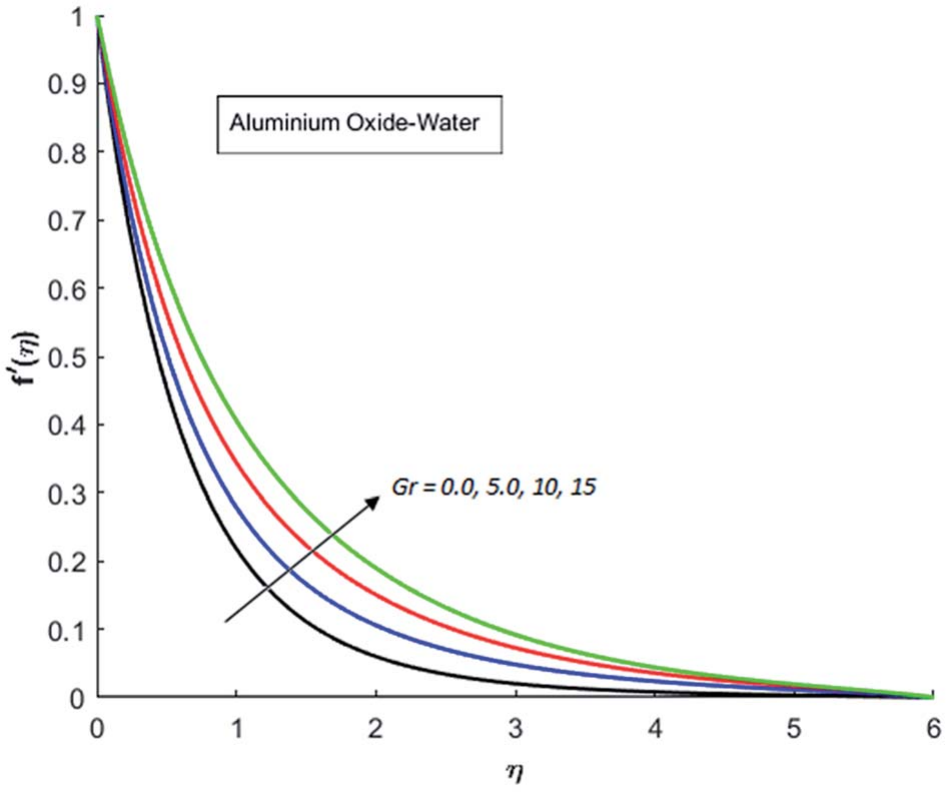
Velocity distribution for various value of Gr when *M* = 1, Pr = 0.3, Ec = 0.1, Nr = 0.2 and *λ* = 1.

**Fig. 8:**
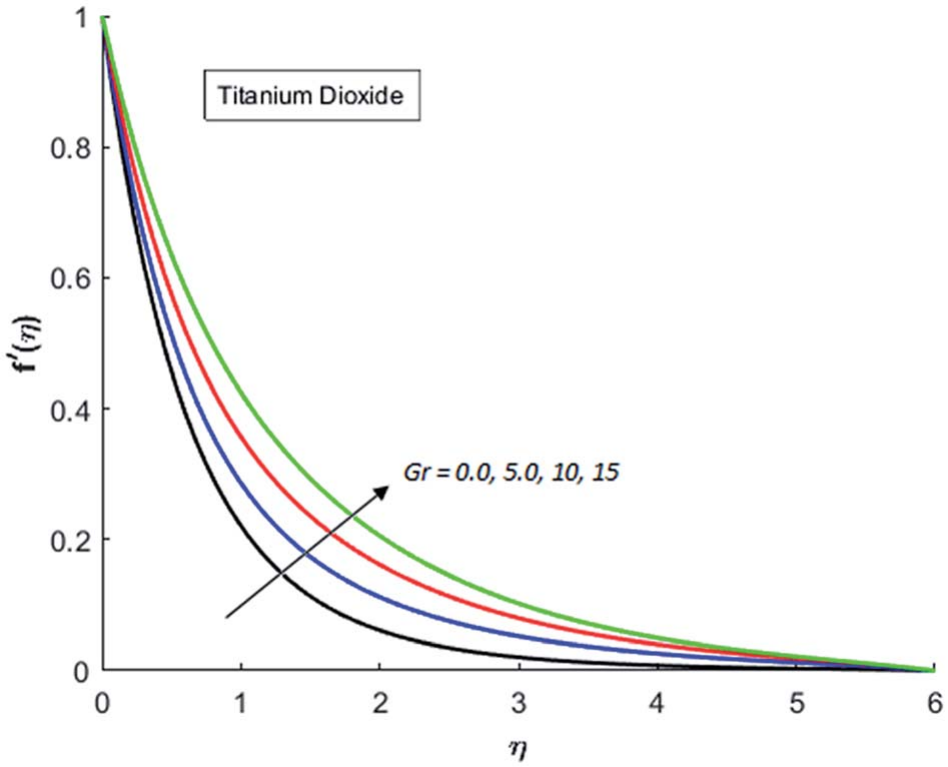
Velocity distribution for various value of Gr when *M* = 1, Pr = 0.3, Ec = 0.1, Nr = 0.2 and *λ* = 1.

**Fig. 9:**
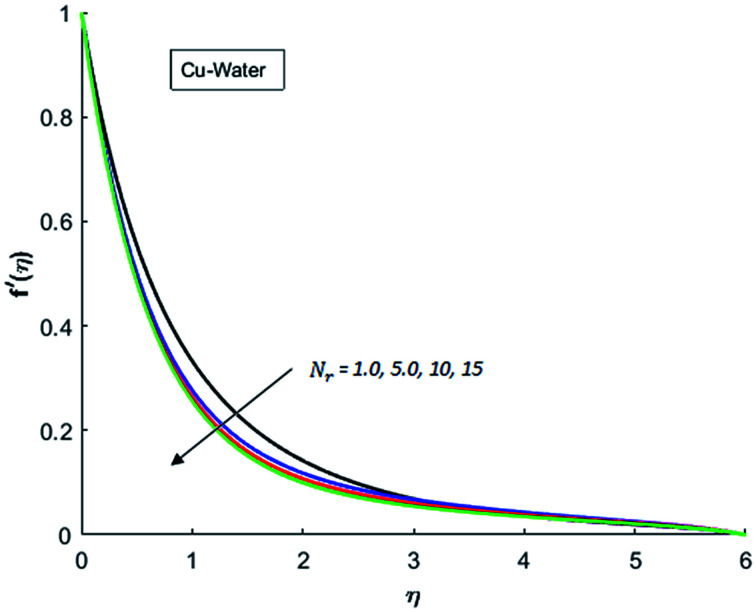
Velocity distribution for various value of Nr when *M* = 0.5, Pr = 0.3, Gr = 0.5, Ec = 0.1 and *λ* = 1.

**Fig. 10:**
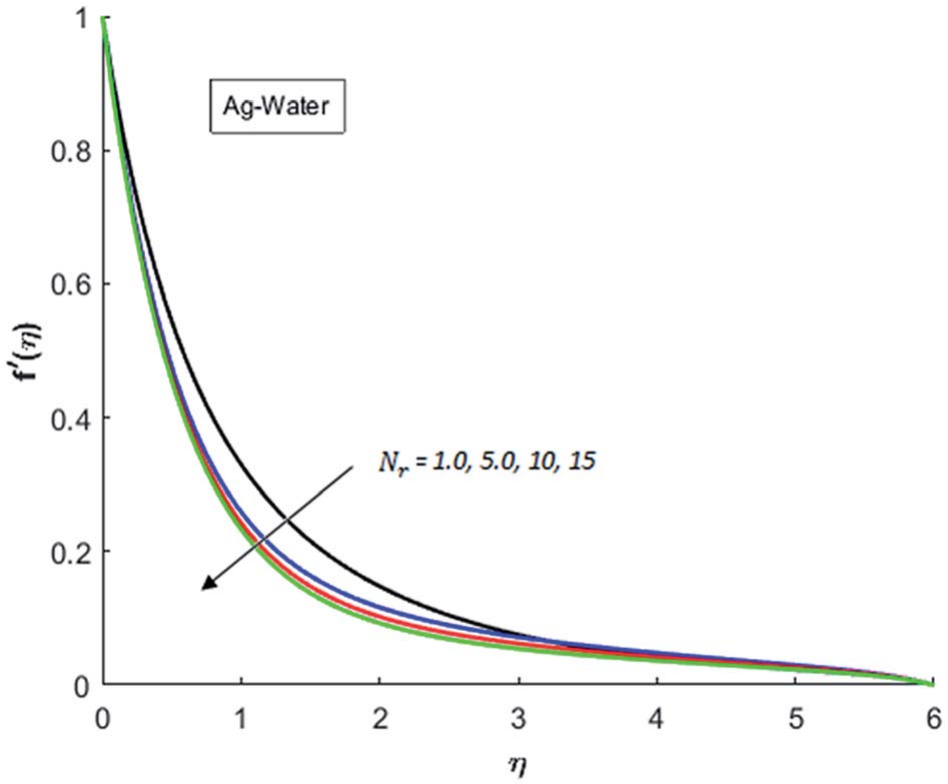
Velocity distribution for various value of Nr when *M* = 0.5, Pr = 0.3, Gr = 0.5, Ec = 0.1 and *λ* = 1.

**Fig. 11:**
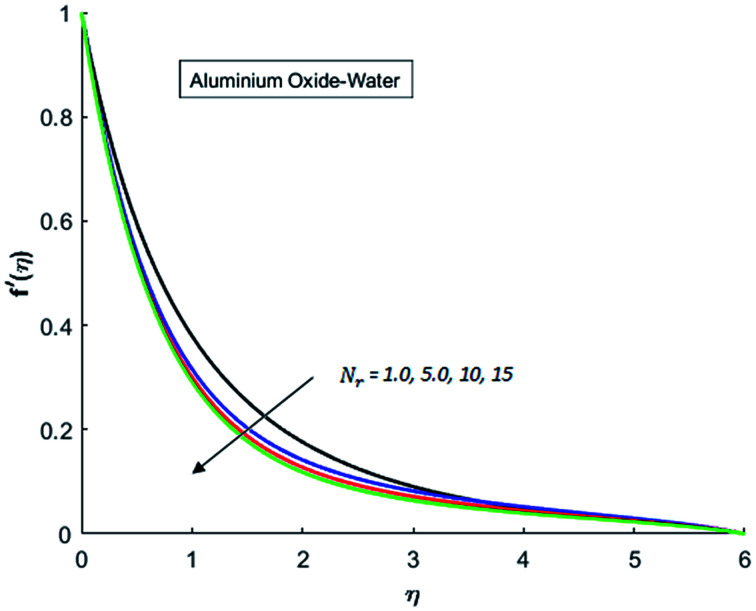
Velocity distribution for various value of Nr when *M* = 0.5, Pr = 0.3, Gr = 0.5, Ec = 0.1 and *λ* = 1.

**Fig. 12:**
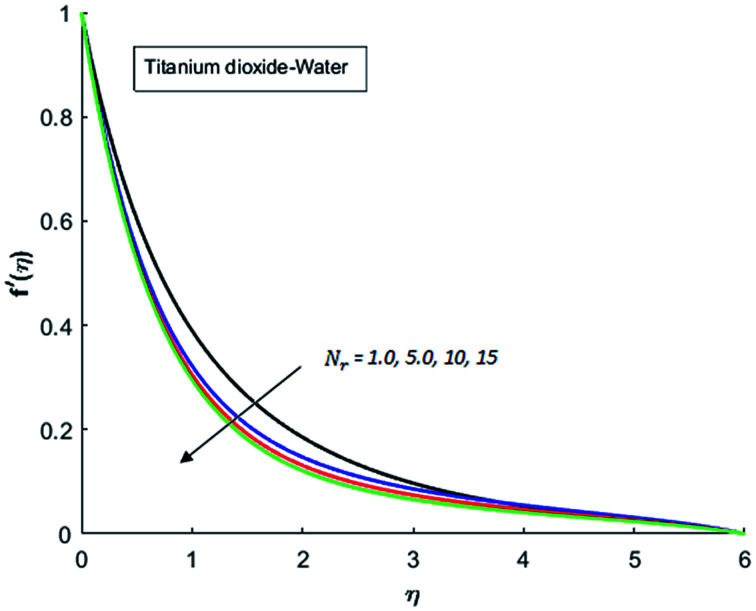
Velocity distribution for various value of Nr when *M* = 0.5, Pr = 0.3, Gr = 0.5, Ec = 0.1 and *λ* = 1.

**Fig. 13:**
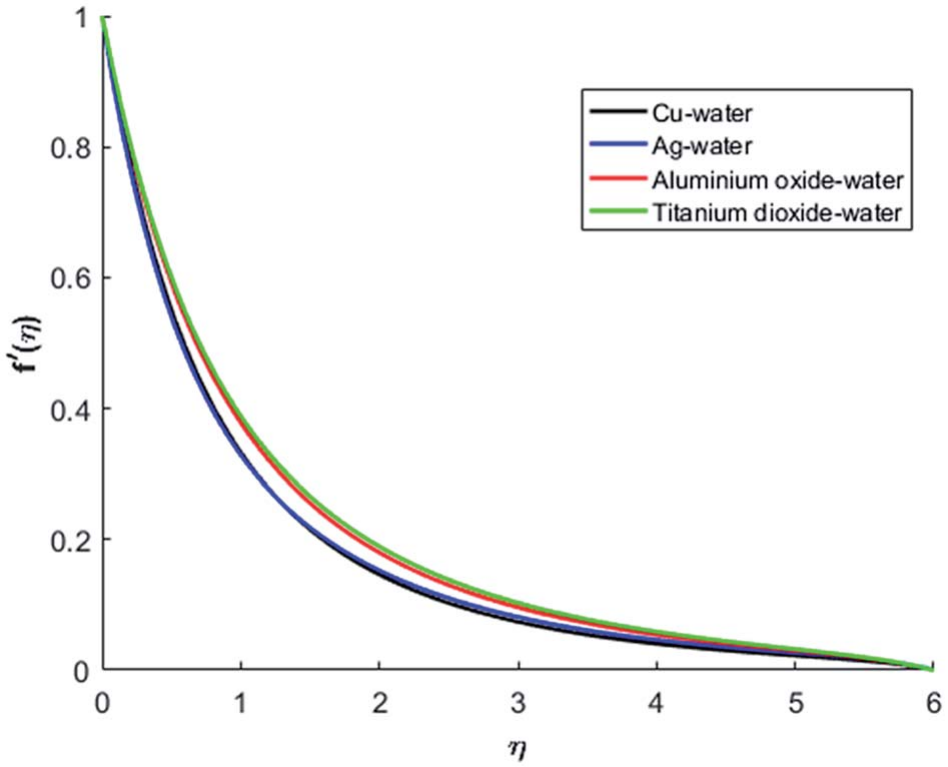
Velocity profiles for different nanofluids when *M* = 0.5, Pr = 0.3, Ec = 0.1, Gr = 5, Nr = 0.2 and *λ* = 1.

Fig. 1–4 display the effects of Eckert number Ec on the dimensional velocity of Cu, Ag, Al_2_O_3_ and TiO_2_-nanofluids when Gr > 0. These figures demonstrate that dimensionless velocity *f*′ increases as Eckert number Ec is increased. Ec is the ratio of kinetic energy to enthalpy and an increase in Ec results an increase in the kinetic energy. This increase in kinetic energy temperature of the fluid rises. This rise in temperature causes density differences which results an increase in the magnitude of buoyancy force. Due to this, as Eckert number is the coefficient of term (in energy eqn (11)) due to Joule heating and an increase in Ec corresponds to an increase in temperature. Grashof number (Gr) is the ratio of buoyancy force to the inertial force and it varies through positive values for downward flow and hence flow is accelerated by gravitational force and therefore, significant increase in the velocity is observed. For evidence, Fig. 5–8 are displayed. Thus by increasing Grashof number (Gr), a significant increase in velocity can be observed from Fig. 5–8. In qualitative sense, the buoyancy force has similar effects on the flow of Cu-nanofluid and TiO_2_-nanofluid. It is also observed that the momentum boundary layer thickness increases when Grashof number (Gr) is increased. During numerical simulations and numerical experiments, it is also noted that the velocity motion of nanofluid decelerates when Gr is varied through negative values. Gr is negative when flow is vertically upward and is opposed by the negative gravity. For the case of negative gravity, fluid motion slows down and a significant reduction in momentum boundary layer thickness is observed for (Gr < 0). The fluid under discussion has a property of emitting thermal radiations in the form of electromagnetic waves. The emission of electromagnetic waves from the fluid regime carries heat energy away which results a significant decrease in the temperature. In order to examine the effects of thermal radiation on the temperature of four types of nanofluids, simulations are carried out and are recorded in Fig. 9–12. It is found from simulations displayed by Fig. 9–12 that the motion of nanofluids slows down due to a reduction in buoyancy force. This is due to the fact that the temperature decreases when Gr > 0. Hence it is concluded that the emission of thermal radiation from the nanofluid of a decrease in the temperature of nanofluid. This decrease in temperature causes a density difference and hence favorable buoyancy force becomes weak. Consequently, fluid motion slows down. It is also observed that momentum boundary thickness is decreased by thermal radiation when gravity is positive. However, opposite behavior is observed for opposing gravitational force. The effects of different nanoparticles on the motion of nanofluids are simulated and are displayed by Fig. 13. This figure reflects that the velocity of Cu-nanofluid is smaller (in magnitude) than the velocities of Ag, Al_2_O_3_ and TiO_2_-nanofluids.

### 4.2 Temperature profiles

**Fig. 14:**
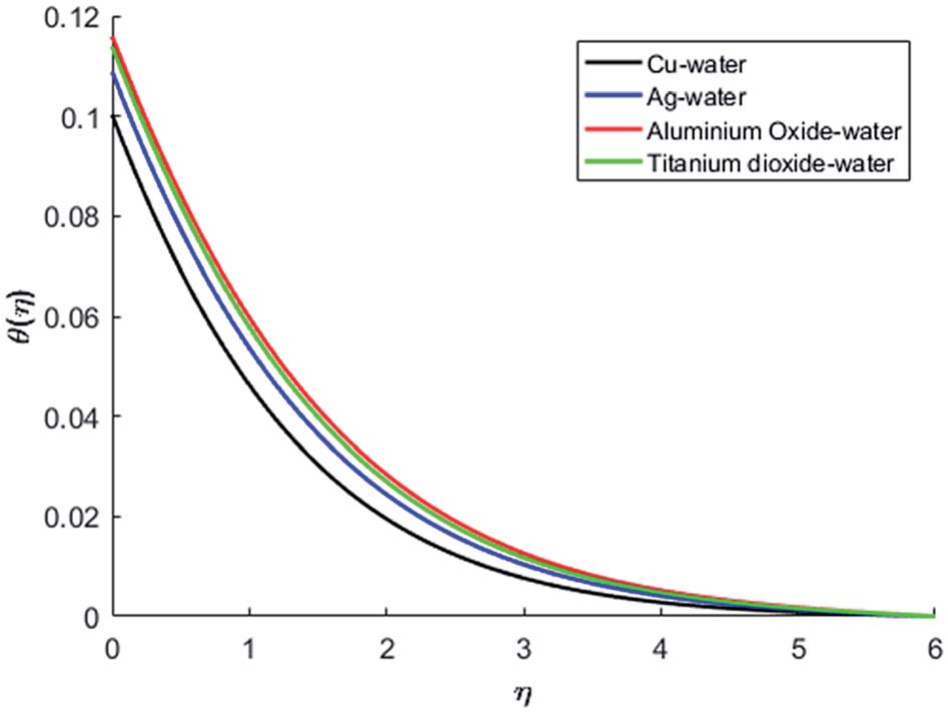
Temperature curves for different nanofluids when *M* = 0.5, Pr = 0.3, Nr = 0.2, Ec = 0.1, Gr = 0.5 and *λ* = 1.

**Fig. 15:**
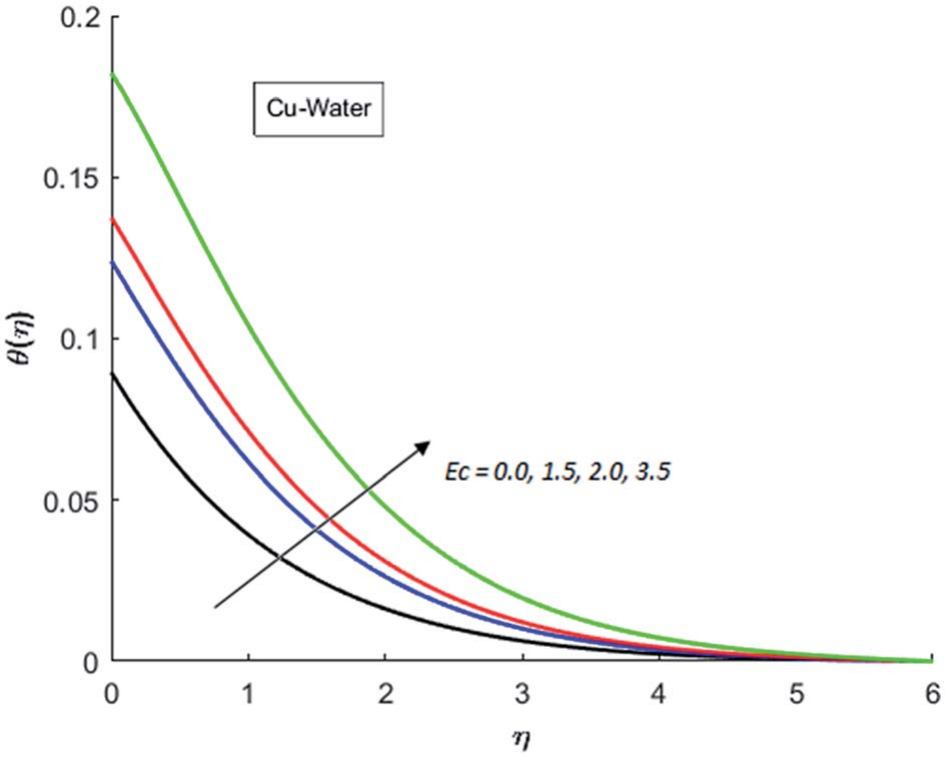
Temperature curves for various value of Ec when *M* = 0.5, Pr = 0.3, Gr = 5, Nr = 0.2 and *λ* = 1.

**Fig. 16:**
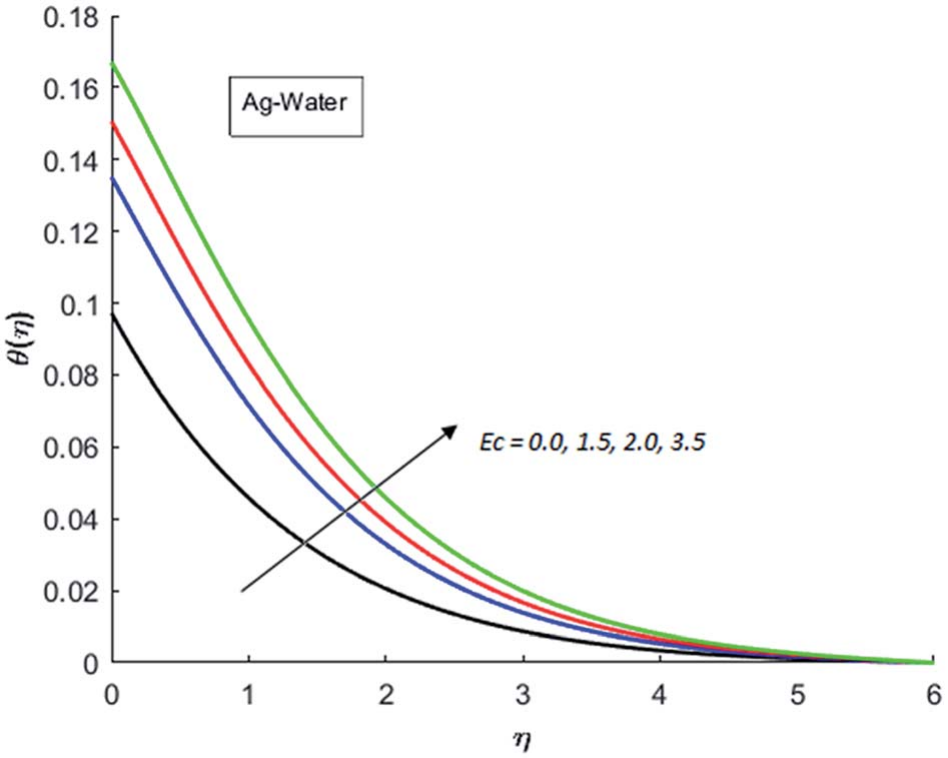
Temperature curves for various value of Ec when *M* = 0.5, Pr = 0.3, Gr = 5, Nr = 0.2 and *λ* = 1.

**Fig. 17:**
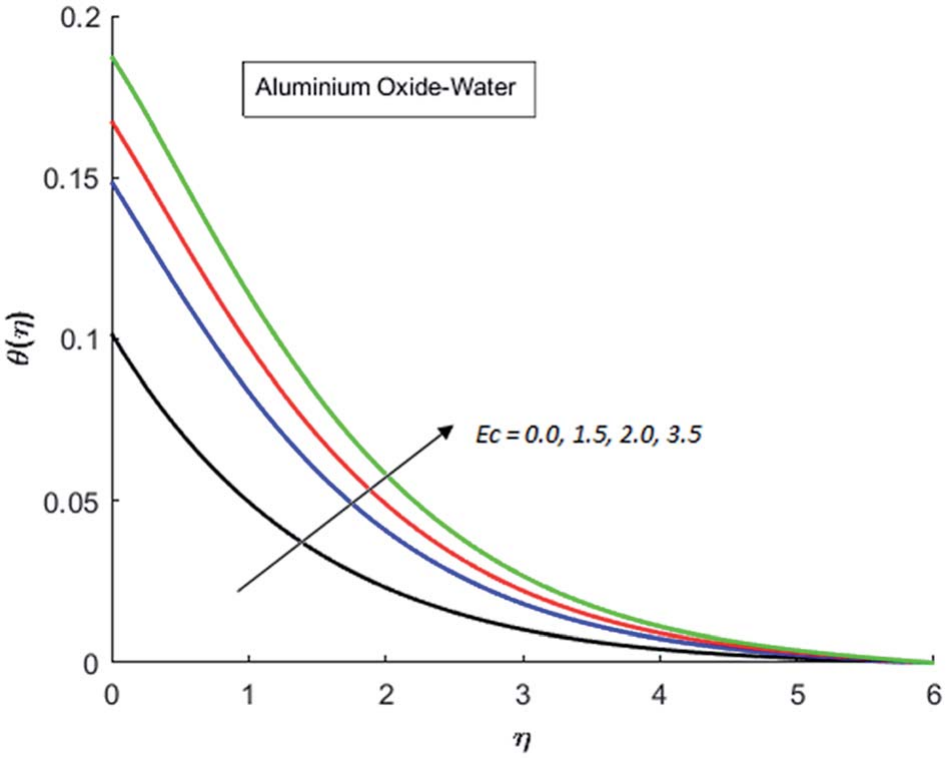
Temperature curves for various value of Ec when *M* = 0.5, Pr = 0.3, Gr = 5, Nr = 0.2 and *λ* = 1.

**Fig. 18:**
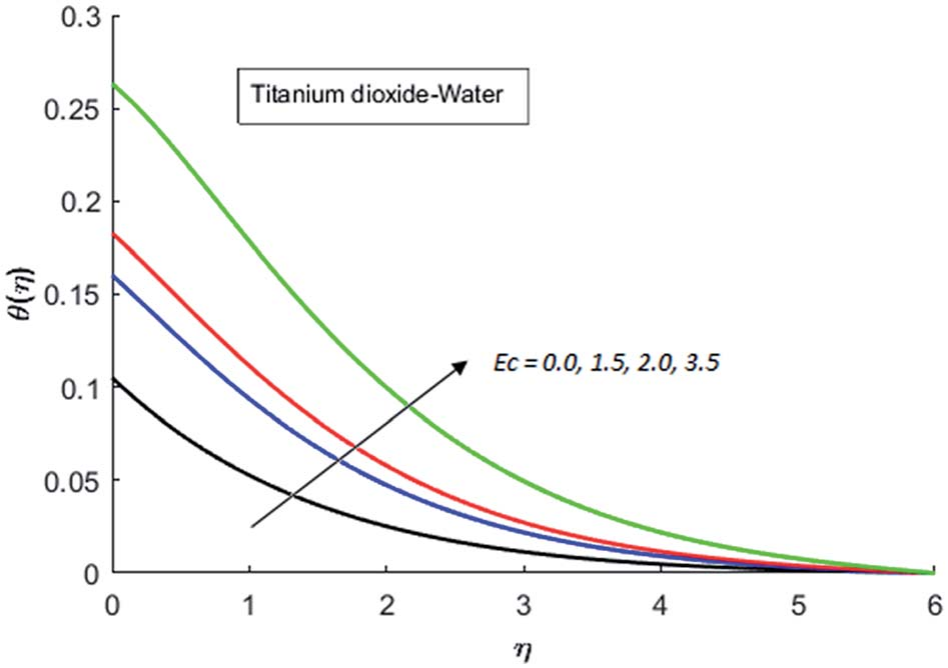
Temperature curves for various value of Ec when *M* = 0.5, Pr = 0.3, Gr = 5, Nr = 0.2 and *λ* = 1.

**Fig. 19:**
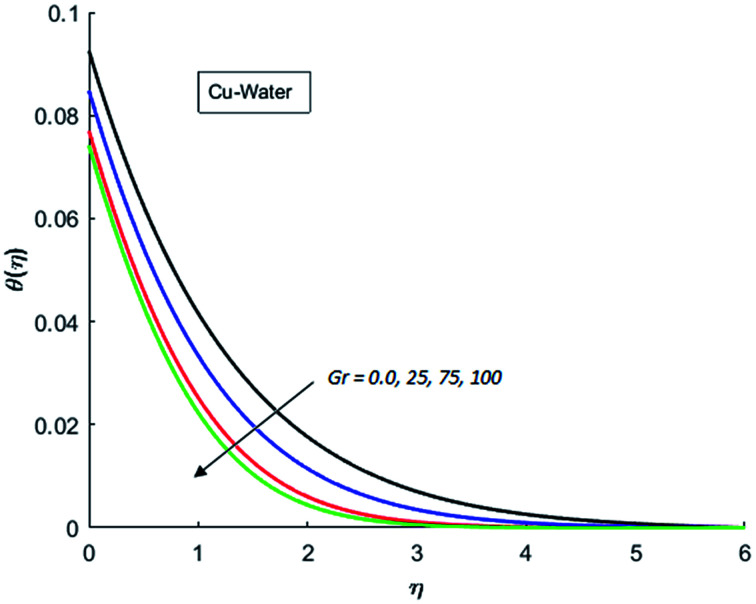
Temperature curves for various value of Gr when *M* = 0.5, Pr = 0.3, Ec = 0.5, Nr = 0.2 and *λ* = 1.

**Fig. 20:**
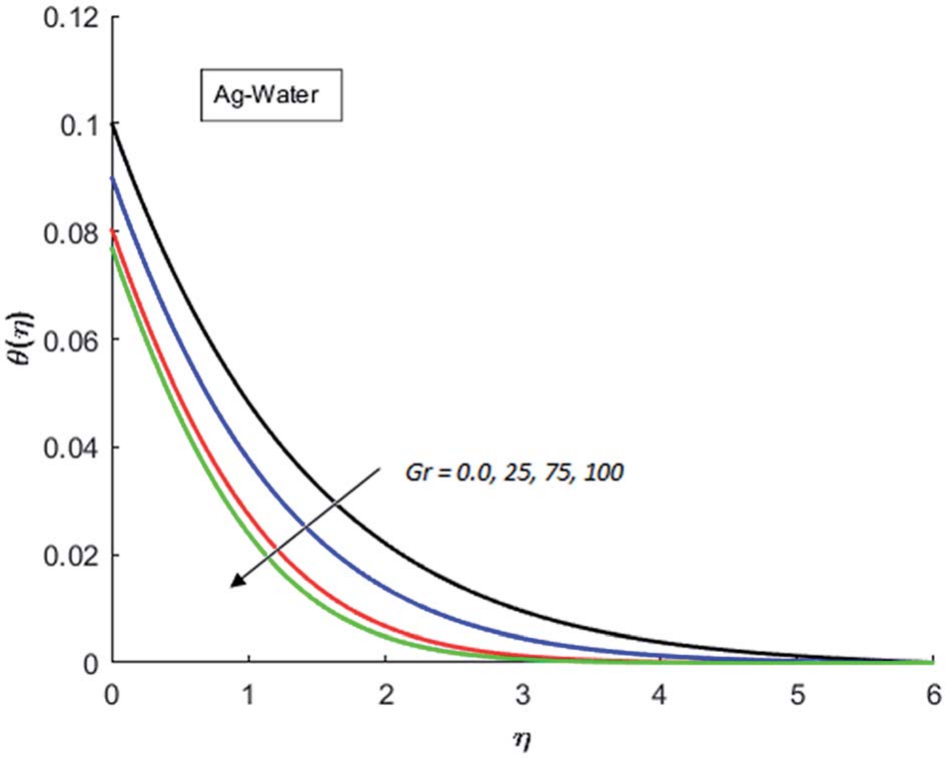
Temperature curves for various value of Gr when *M* = 0.5, Pr = 0.3, Ec = 0.5, Nr = 0.2 and *λ* = 1.

**Fig. 21:**
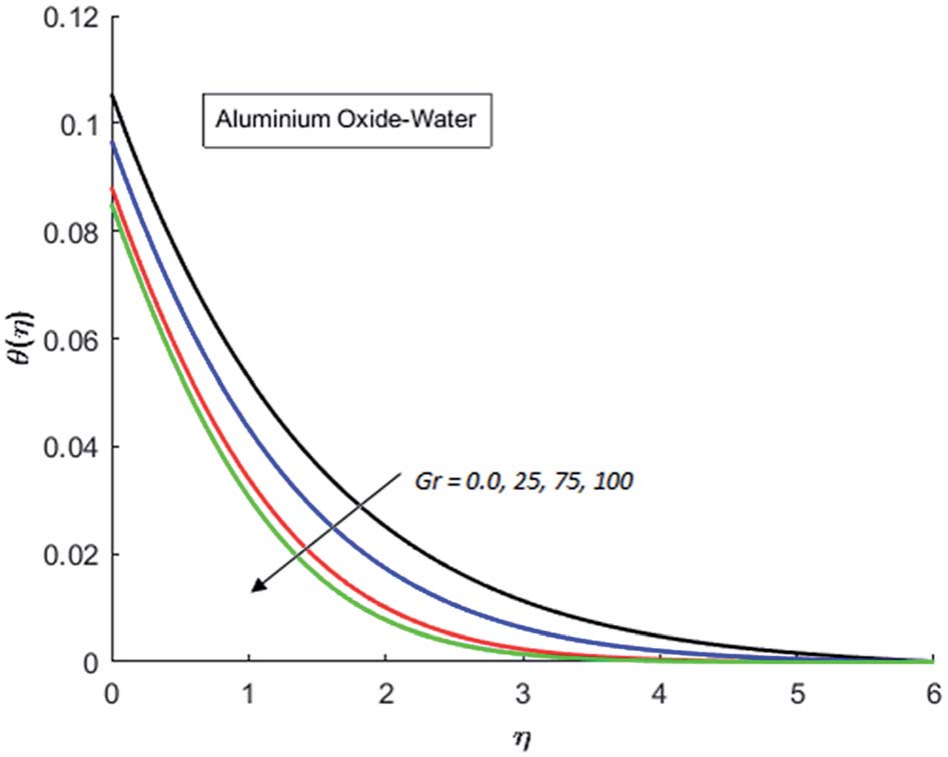
Temperature curves for various value of Gr when *M* = 0.5, Pr = 0.3, Ec = 0.5, Nr = 0.2 and *λ* = 1.

**Fig. 22:**
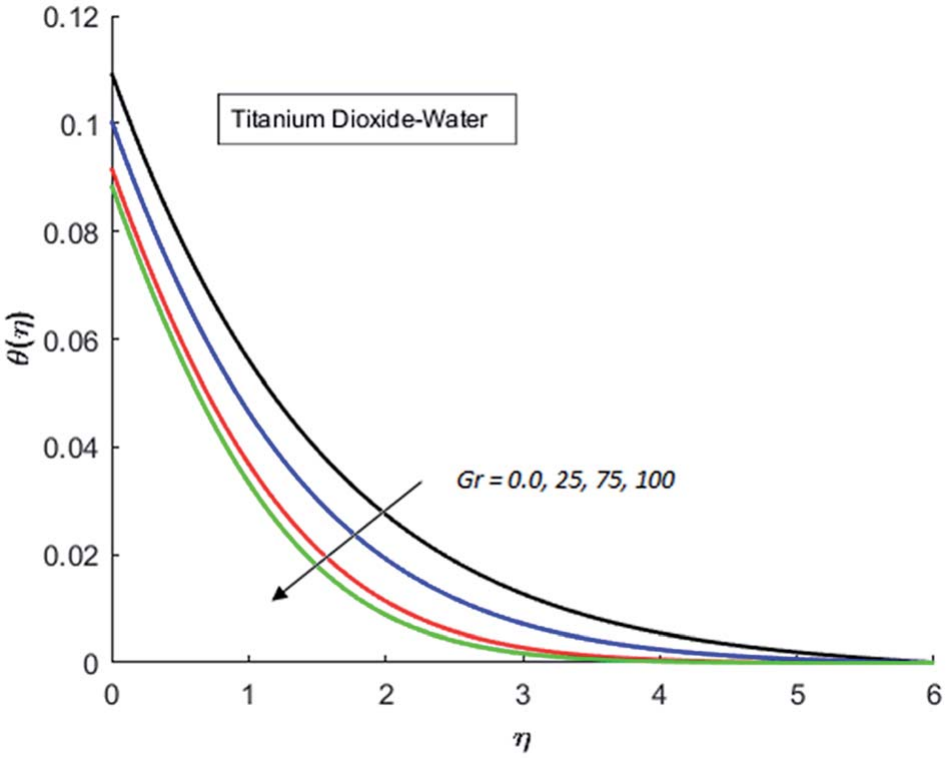
Temperature curves for various value of Gr when *M* = 0.5, Pr = 0.3, Ec = 0.5, Nr = 0.2 and *λ* = 1.

**Fig. 23:**
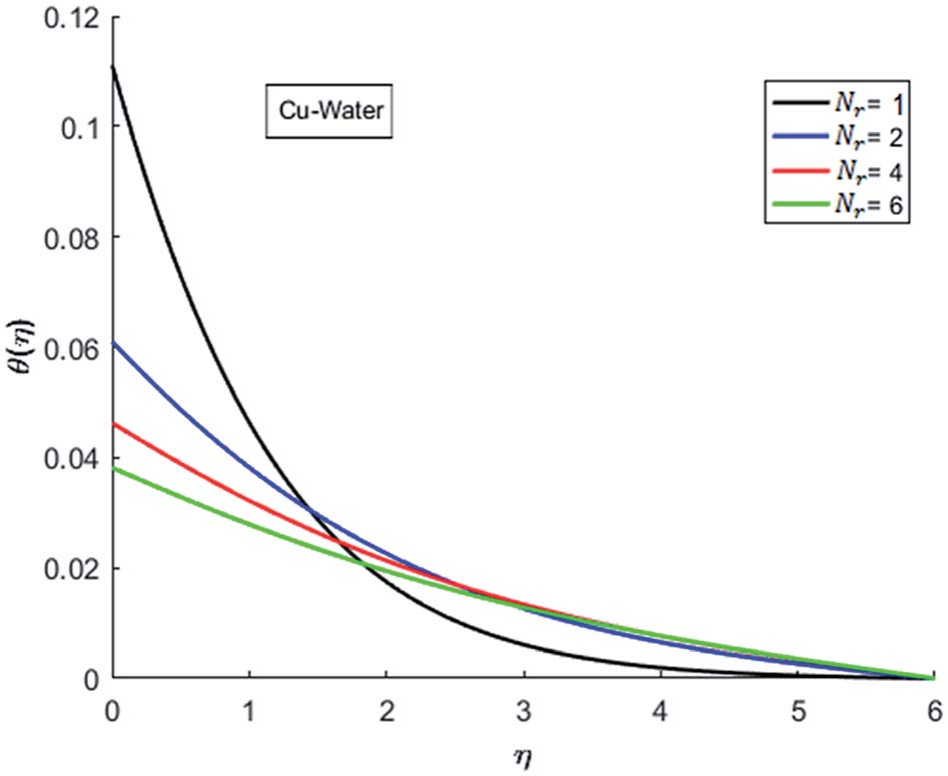
Temperature curves for various value of Nr when *M* = 0.5, Pr = 0.3, Ec = 0.1, Gr = 0.5 and *λ* = 1.

**Fig. 24:**
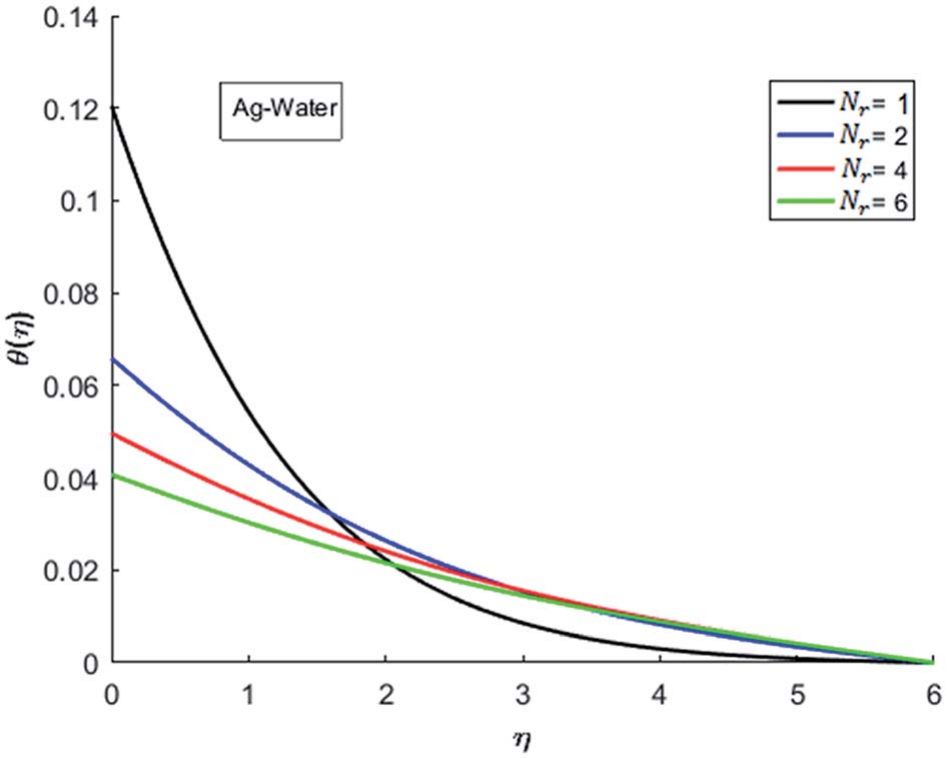
Temperature curves for various value of Nr when *M* = 0.5, Pr = 0.3, Ec = 0.1, Gr = 0.5 and *λ* = 1.

**Fig. 25:**
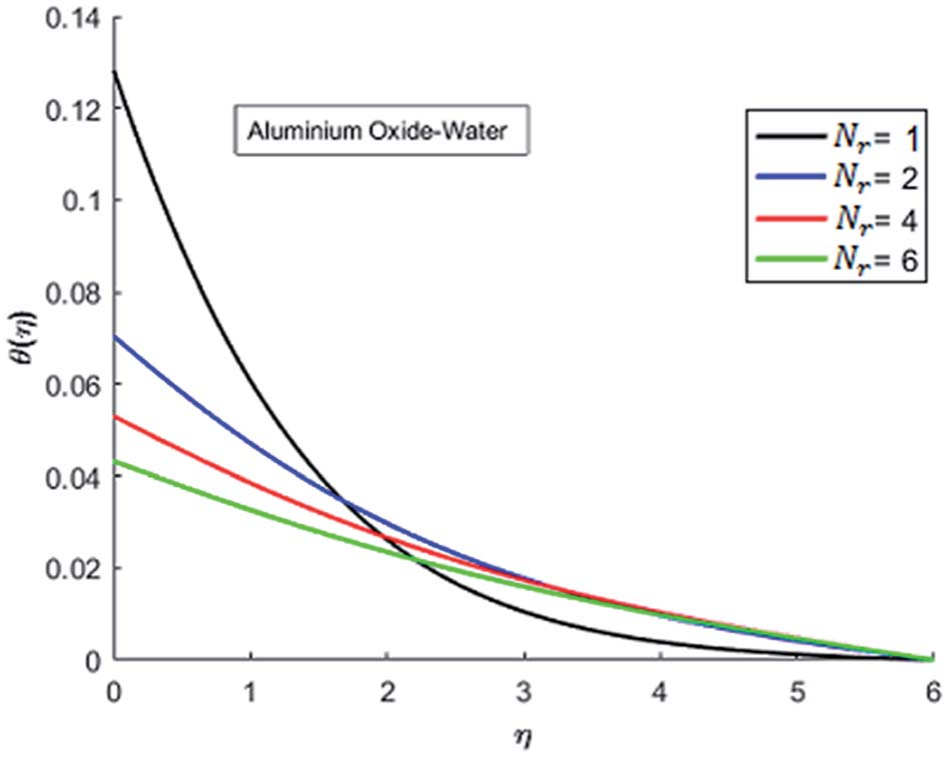
Temperature curves for various value of Nr when *M* = 0.5, Pr = 0.3, Ec = 0.1, Gr = 0.5 and *λ* = 1.

**Fig. 26:**
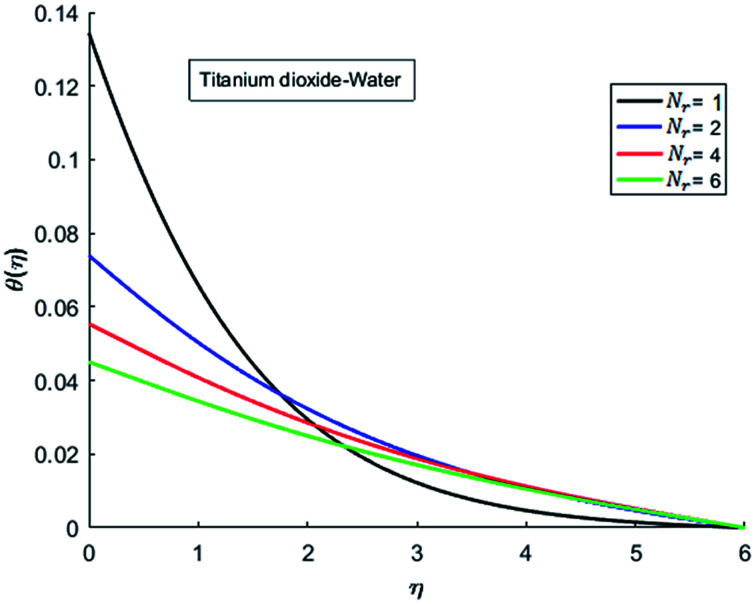
Temperature curves for various value of Nr when *M* = 0.5, Pr = 0.3, Ec = 0.1, Gr = 0.5 and *λ* = 1.

The effects of dispersion of nanoparticles (Cu, Ag, Al_2_O_3_ and TiO_2_) buoyancy force and thermal radiations on the transport of heat in the flow of nanofluid are simulated and results are displayed by the Fig. 14–26. Respectively, Fig. 14 depicts that the temperature of Al_2_O_3_-nanofluid is high as compare to the temperature of Cu, Ag and TiO_2_-nanofluids and *vice versa* for Cu-nanofluid. The effects of Joule heating phenomenon on the temperature of four types of nanofluids are displayed in Fig. 15–18. These figures reflects that the temperature of the nanofluids increases as Eckert number Ec is increased. This increase in temperature (for four types of nano-particles) is due to the fact that Ec and *M* are the coefficient of Joule heating term in the dimensionless form of energy equation. An increase in Ec means that the effect of Joule heating becomes more and more strong and correspond to the generation of more heat due to ohmic dissipation of the fluid. Consequently, this heat adds to the fluid and hence temperature rises. Comparative study of Fig. 15–18 also shows that in TiO_2_-nanofluid highest amount of heat dissipates. It is already mentioned that the three types of modes of heat transfer (convection, conduction and thermal radiation) are considered. Further, opposing and favorable buoyant force is considered. In case of positive buoyant force (Gr > 0), flow experiences a favorable force due to which convection phenomenon becomes significant and process of carrying heat from hot wall to the fluid speeds up. Hence temperature of the fluid rises (see figures). This fact is completely in agreement with the physics of fluid flow (see Fig. 19–22). The four type of nanoparticles are dispersed in the fluid which is capable of radiating heat in the form of the electromagnetic waves as heat passes through it. Here in this study the effect of radiative nature is examined through radiation parameter Nr. An increase in the radiation parameter Nr represents the situation for which more electromagnetic waves carry heat energy away from the fluid. That is why temperature of nanofluid (four types of nanofluids) decreases with an increase in the radiation parameter Nr as shown in Fig. 23–26.

### 4.3 Entropy analysis

The entropy generation due to temperature gradient, viscous dissipation and Joule heating is defined by
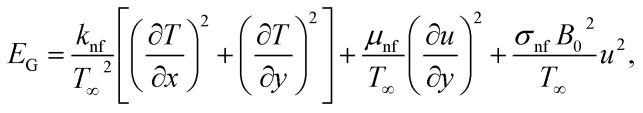


Using the similarity transformations given in eqn (8), one obtains the following dimensionless form of the entropy generation
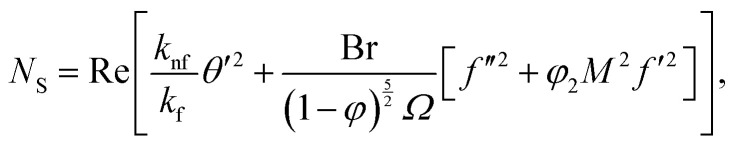
where,
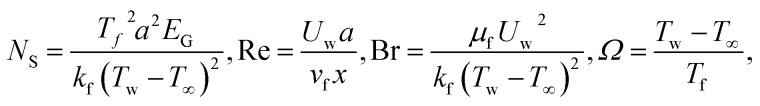
and, respectively, are called dimensionless the entropy generation number, the Reynolds number, the Brinkman number and the non-dimensionless temperature difference number.

### 4.4 Entropy generation profiles

**Fig. 27:**
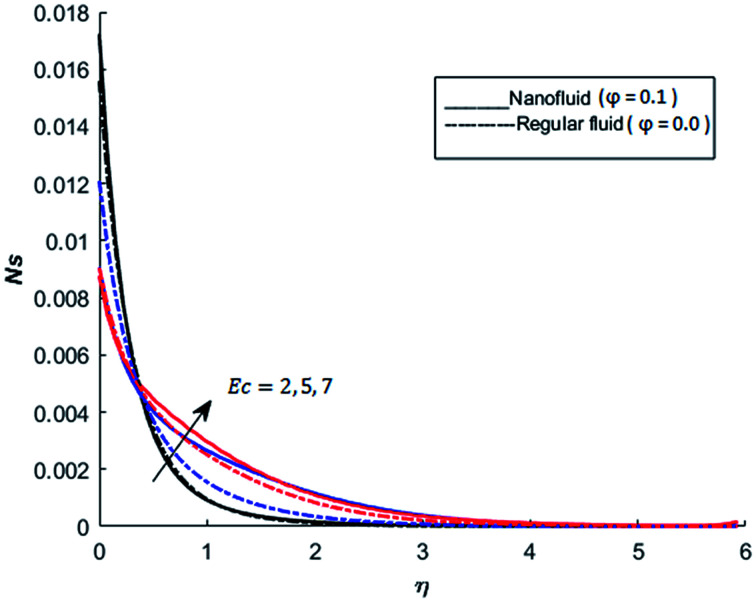
The effect of Eckert number Ec on the entropy generation when Pr = 0.3, *M* = 1, Bi = 0.1, Nr = 0.2, Gr = 5, *λ* = 1, Re = 1, Br = 1 and *Ω* = 1.

**Fig. 28:**
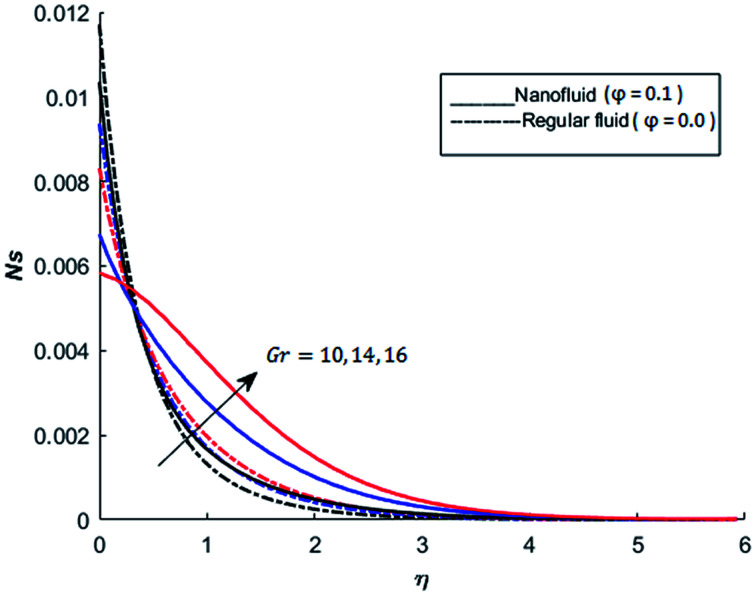
The effect of Grashof number Gr on the entropy generation when Pr = 0.3, *M* = 1, Bi = 0.1, Nr = 0.2, Ec = 0.5, *λ* = 1, Re = 1, Br = 1 and *Ω* = 1.

**Fig. 29:**
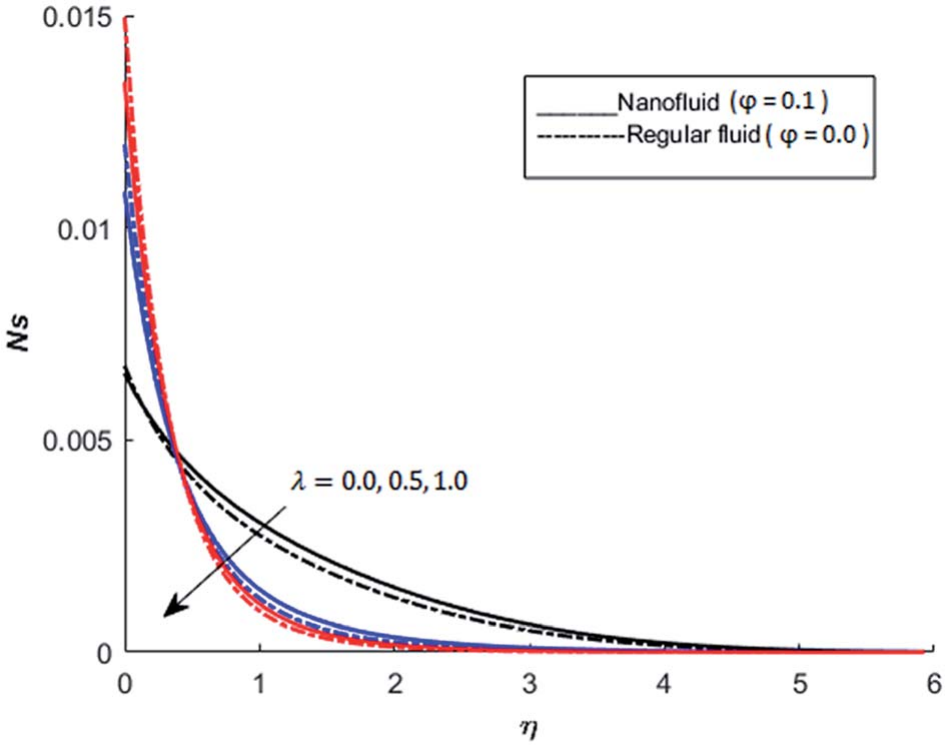
The effect of *λ* on the entropy generation when Pr = 0.3, *M* = 1, Bi = 0.1, Nr = 0.2, Gr = 5, Ec = 0.5, Re = 1, Br = 1 and *Ω* = 1.

**Fig. 30:**
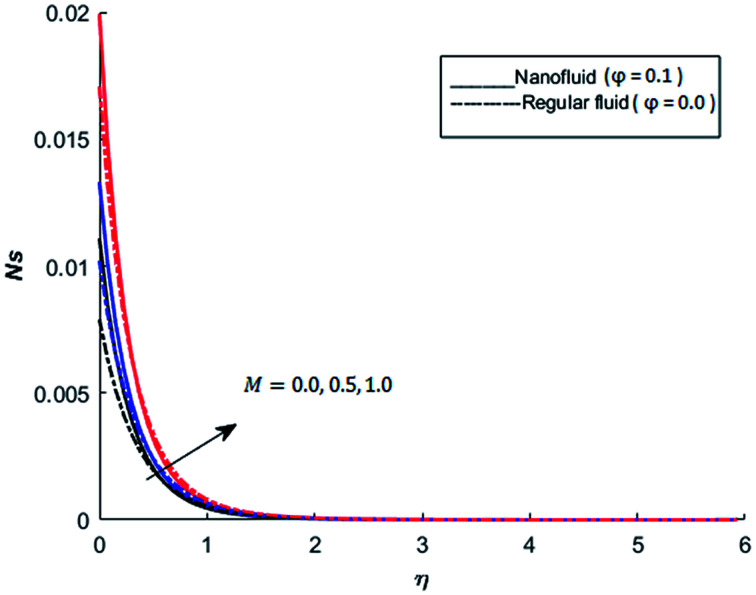
The effect of *M* on the entropy generation when Pr = 0.3, Bi = 0.1, Nr = 0.2, Gr = 5, Ec = 0.5, *λ* = 1, Re = 1, Br = 1 and *Ω* = 1.

**Fig. 31:**
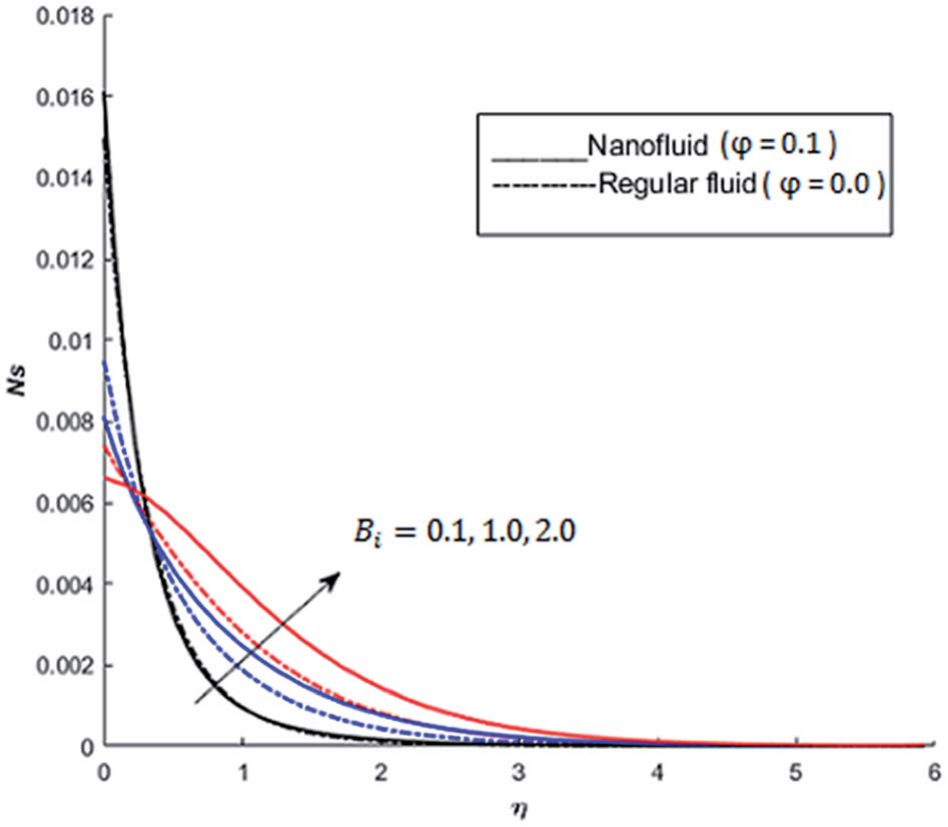
The effect of Biot number Bi on the entropy generation when Pr = 0.3, Nr = 0.2, Gr = 5, Ec = 0.5, *λ* = 1, Re = 1, Br = 1 and *Ω* = 1.

The behavior of dimensionless entropy under the variation of Eckert number Ec, Grashof number Gr, unsteadiness parameter *M* and Biot number Bi is displayed in Fig. 27–31. Fig. 27 represents that rate of the entropy generation increases when Ec is increased. Therefore, it can be advised to use the fluid exhibiting less dissipation in order to avoid losses of heat energy in magneto-thermal system. This recommendation is both for nano and pure fluid. Despite of the advantage of magnetic fluid to control the momentum boundary layer thickness, it is not recommended to use electrically conducting fluid when the reduction of losses of heat energy is of high concern. The effect of buoyancy force on the entropy generation is also simulated and the simulated results are graphed in Fig. 28. This figure reflects that favorable buoyancy force causes an increase in the energy losses. These losses can be controlled by introducing the opposing buoyancy force *i.e*. considering downward flow on vertical sheet. Fig. 28 also demonstrates that losses of heat energy are significant for nanofluid as compare to the pure fluid. The entropy generation in steady and unsteady flow of both nanofluids and regular fluids is represented in Fig. 29. It is noted from Fig. 29 that the entropy generation is high in steady flow as compare to unsteady flow. The effect of Joule heating on the entropy generation is displayed in Fig. 30. This figure depicts that there is significant increase in the entropy generation when heat losses due to dissipation caused by the external magnetic field. This behavior is same for both pure and nanofluid. Therefore, it is advised not to use electrically conducting fluid. Alternatively, magnetic intensity of the fluid be adjusted in such a way that losses of heat energy should be minimum. This is for both nano and regular fluids. The entropy generation in nano-magnetohydrodynamic flow is high as compare to nano-hydrodynamic flow (see Fig. 30). The effect of convection boundary condition on the entropy generation is displayed in Fig. 31 is noted from this figure shows that there is a significant effect of Biot number (dimensionless number due to convective boundary conduction) on the entropy generation.

## 5 Conclusion

In this paper, the effects of four types of nano-particles (Cu, Ag, Al_2_O_3_ and TiO_2_) on the transport of heat in unsteady two-dimensional boundary layer flow of a radiative fluid over a convectively heated surface in the presence of Joule heating, heat absorption/generation and buoyant force are investigated. It is observed that dispersion of nano-particles in the pure fluid increases the thermal conductivity of the resulting mixture which may play a vital role in the thermal systems. For favorable buoyant force the velocity of the mixture (mixture of nanoparticles and radiative fluid) increases which causes an increase in the thermal and momentum boundary layer thicknesses. However, in case of opposing buoyant force, a reverse mechanism regarding momentum and thermal boundary layer thicknesses is observed. The magnetic field intensity and ohmic dissipation are directly proportional with each other. Hence an increase in the intensity of the magnetic field converts more electrical energy into heat (due to ohmic dissipation process). It is also observed that an increase in the intensity of the magnetic field retards the flow and reduces the momentum boundary thicknesses. Therefore, it is advised that an external magnetic field may be applied to control the flow and momentum boundary layer thickness. However, it should be in mind that an increase in the imposition of external magnetic field has opposite effect on the thermal boundary layer thicknesses due to Joule heating mechanism. It is also important to mention that momentum boundary layer thickness for hydrodynamic flow is higher than that of the magnetohydrodynamic flow. However, thermal boundary layer thickness of hydrodynamic flow is less than that of the magnetohydrodynamic flow. During numerical computations, it is studied that the velocity of TiO_2_-nanofluid is higher than the velocity of Al_2_O_3_, Ag and Cu-nanofluids. Due to magnetic field and fluid flow interactions, the electrical energy converts into heat. This may undesirable in many thermal systems. Therefore, control of Joule heating in the design of thermal system is necessary. However, this dissipation of heat may be desirable in some biological fluid flows. Moreover, an increase in the intensity of the magnetic field causes an increase in the entropy generation. The positive buoyancy force enhances the entropy generation. However, opposing buoyancy force reduces energy losses. Energy losses in steady flow are high as compare to the unsteady flow. The key observations are listed below:

• The buoyant force is responsible for the influence of thermal radiations on the flow of nanofluid. It is observed that if buoyant force is not considered, then there is no effect of thermal radiations on the flow and hence momentum boundary layer thickness. As the buoyant force is significant in vertical flows, therefore, it is recommended that horizontal arrangement of physical model (sheet) should be taken if no impact of thermal radiations on the flow of nanofluid is desired.

• The magnetic field decelerates the fluid motion due to hindrance caused by the Lorentz force. Therefore, it is recommended to apply external magnetic field perpendicular to the plane of sheet if momentum boundary layer thickness is to be controlled.

• Convectively heated surface causes more entropy generation. Therefore, it is recommended not to use the convectively heated surface in thermal systems.

• Imposition of external magnetic field increases the entropy generation and is responsible of great energy loses. Therefore, thermal systems work efficiently without loses of energy if external magnetic field is not imposed.

## Conflicts of interest

There are no conflicts to declare.

## Supplementary Material
